# A non-hypothesis-driven practical laboratory activity on functional metagenomics: “fishing” protein-coding DNA sequences from microbiomes

**DOI:** 10.3389/fbioe.2025.1602982

**Published:** 2025-05-20

**Authors:** Melissa Morra, Denise Marradi, Luca Gandini, Vittorio Ivagnes, Giulia Ottolini, Alessandro Bovio, Grace Jabali, Lorenzo Maraschi, Ifeoluwa Ayomide Dada, Tonderai Vitalis Chawanda, Martina Gorla, Olga Tarasiuk, Chiara Mocchetti, Maria Felicia Soluri, Francesca Boccafoschi, Daniele Sblattero, Diego Cotella

**Affiliations:** ^1^ Department of Health Sciences, University of Eastern Piedmont, Novara, Italy; ^2^ Research Center on Autoimmune and Allergic Diseases (CAAD), University of Eastern Piedmont, Novara, Italy; ^3^ Department of Life Sciences, University of Trieste, Trieste, Italy

**Keywords:** open reading frame, domainome, microbiome, course-based undergraduate research experience, synthetic biology

## Abstract

Practical laboratory of the most functional metagenomics courses focuses on activities aimed at providing specific skills in bioinformatics through the analysis of genomic datasets. However, sequence-based analyses of metagenomes should be complemented by function-based analyses, to provide evidential knowledge of gene function. A “true” functional metagenomic approach relies on the construction and screening of metagenomic libraries - physical libraries that contain DNA cloned from metagenomes of various origin. The information obtained from functional metagenomics will help in future annotations of gene function and serve as a complement to sequence-based metagenomics. Here, we describe a simple protocol for the construction of a metagenomic DNA library, optimized and tested by a team of undergraduate biotechnology students. This protocol is based on a technique developed in our laboratory and currently used for research. Using this protocol, libraries of protein domains can be quickly generated, from the DNA of any intron-less genome, such as those of bacteria or phages. Therefore, these libraries provide a valuable platform for training students in various validation tools, including computational methods - for example, metagenome assembly, functional annotation - and proteomics techniques, including protein expression and analysis. By varying the biological source and validation pipeline, this approach offers virtually limitless opportunities for innovative thesis research projects.

## 1 Introduction

Functional genomics is a discipline which, by combining Bioinformatics, Next-Generation Sequencing (NGS) and other Omics technologies, aims to assign functions and interactions to genes and their products of expression (Hieter and Boguski, 1997; Caudai et al., 2021). Several universities and research institutes offer hands-on courses on NGS and Bioinformatics as part of their advanced genomics-related courses.

Metagenomics has added a new level of complexity by allowing scientists to study the entire microbial population (or “microbiota”) of a specific environment—like soil, the ocean, skin, or hot springs—at the genomic level. To give an example, the human gut microbiome (the collection of the genomes of all microorganisms living in the gut) records at least 3.3 million unique genes, 150 times more genes than our genome, because of a community of about 1,000 bacterial species that cohabit in our intestine (Qin et al., 2010). To provide knowledge of gene function, sequence-based analyses of metagenomes must be complemented by function-based analyses, for example, enzymatic assays (Wiltschi et al., 2020).

Incorporating functional metagenomics into university-level Life Science education offers advantages not only for students but also for researchers and their work. Research has shown that when researchers teach, it enhances their understanding, prompting students to ask new and often unexpected questions. This process can lead to fresh research directions and drive the development of innovative solutions to complex problems (Jurkowski et al., 2007). If the biology community can integrate functional metagenomics education with ongoing research advancements from the outset, students could play an active role in advancing the field. Teaching a new or emerging area of study is an excellent way to engage students in addressing key scientific questions and inspiring them to pose their own inquiries. In the case of functional metagenomics, even the simplest questions can yield profound insights. Answering these questions benefits both emerging scientists and established researchers. Many initiatives are currently underway to merge (meta) genomics research with education (Muth and Caplan, 2020; Ginnan and Bordenstein, 2023; Fuhrmeister et al., 2021; Heller et al., 2024).

The principle underlying Functional Metagenomics is to isolate DNA from microbial communities and to clone it into a suitable host (for example, *Escherichia coli*); each clone harbours a fragment (usually 25–40 kb in size) of the DNA isolate. Then, the metagenomic DNA library undergoes a screening process specifically designed to identify those clones with a desired activity - for example, antibiotic resistance, ability to catalyse a specific chemical reaction, bactericidal (Lam et al., 2015; Berini et al., 2017). This function-based approach enables the discovery of novel proteins whose functions would not be predicted based on DNA sequence alone.

**FIGURE 1:**
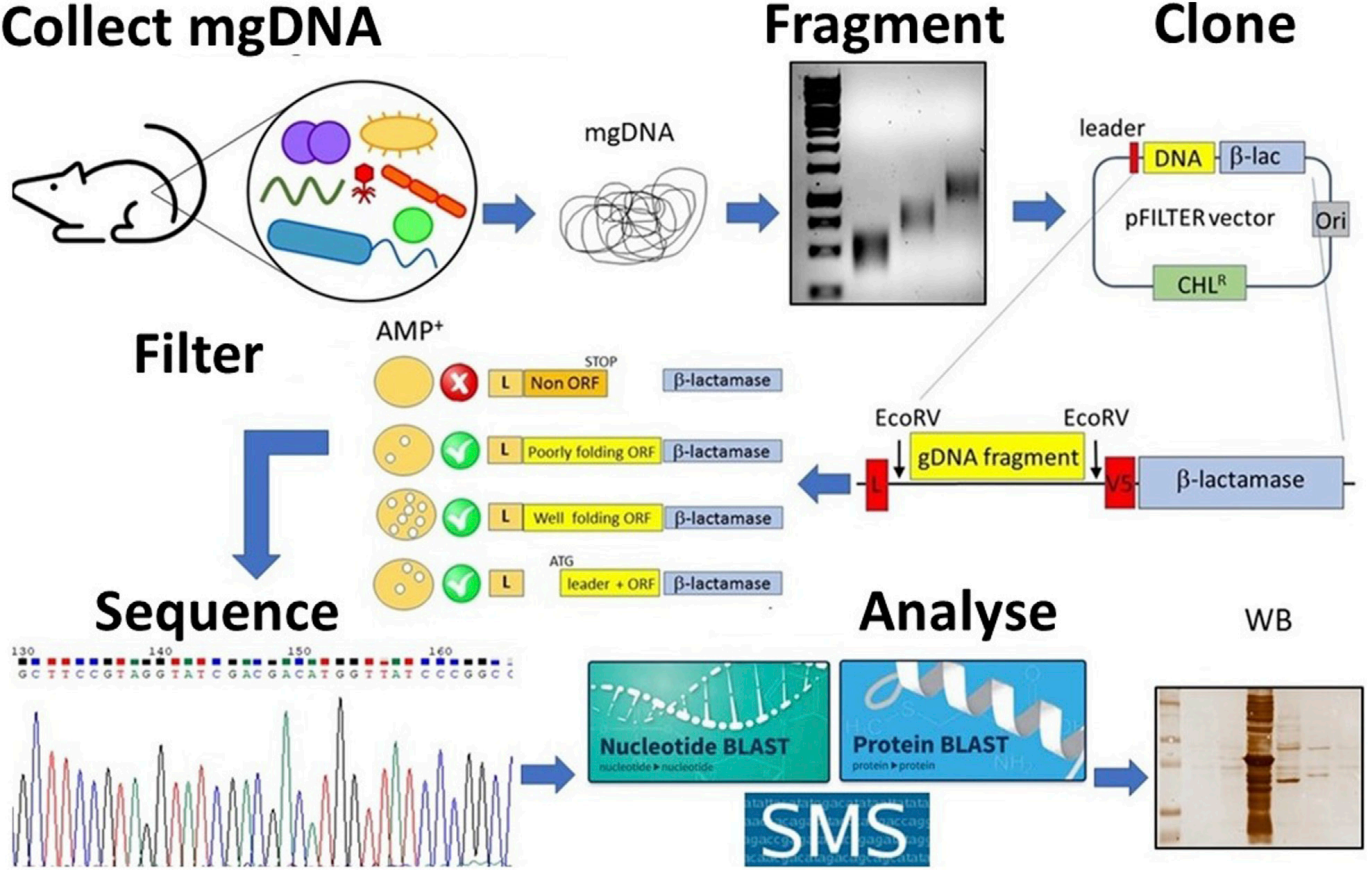
Schematic overview of the student’s project. Metagenomic DNA (mgDNA) is extracted from an appropriate source (such as murine feces), then fragmented into a collection of 250–1,000 bp fragments and cloned into the pFILTER vector. *E. coli* cells are transformed with the ligation product and plated on ampicillin-containing media to carry out the “filtering” of open reading frames (ORFs). The resulting clones are sequenced and analyzed using computational tools (for example, BLAST, AlphaFold) as well as functional assays (for example, Western blotting, enzyme assays).

Although seemingly simple, this procedure involves many steps, which makes the construction of metagenomic libraries laborious and time-consuming, requiring a high level of skills at the laboratory bench (Terrón-González et al., 2014; Lam et al., 2015). Moreover, this process has many other limitations such as the poor expression of correctly folded full-length proteins in heterologous hosts (Pouresmaeil and Azizi-Dargahlou, 2023). To simplify the production of metagenomic DNA libraries and to overcome the limitations associated, we have developed an approach aimed at “filtering” genomic DNA to generate expression libraries enriched in functional protein domains. This approach is based on the knowledge that >85% of the genome of prokaryotes is translated into proteins (Land et al., 2015) and that most proteins are organized into multiple domains, evolutionarily conserved, each of them contributing to a distinct function (Heger and Holm, 2003). Based on bioinformatic analyses indicating that the most common domain size is approximately 100 amino acids, any piece of prokaryotic DNA >150 nucleotides (50 amino acids) is likely to encode a protein domain (Tiessen et al., 2012). The recovery of functional open reading frames (ORFs) from bacterial DNA may therefore be a straightforward procedure (D’Angelo et al., 2011; Soluri et al., 2018). In brief, genomic DNA is randomly fragmented into short (250–1,000 nucleotides) fragments, cloned between a secretory leader sequence (a signal peptide) and the ß-lactamase gene in the pFILTER plasmid (Soluri et al., 2018), and transformed into *E. coli*. Transformed bacteria are then seeded on ampicillin-containing agar plates, and only those clones harbouring an ORF properly folded and in the correct frame with both the signal peptide and the ß-lactamase will grow under selective pressure (Figure 1). So, if the protein is functional, it can restore the activity of the gene which enables the *E. coli* to resist the ampicillin and grow. Such expression libraries of protein domains (the “*domainome*”) will be useful for many purposes, including structural studies, antibody generation, protein/substrate binding analyses, domain shuffling for enzyme evolution and protein arrays (Gourlay et al., 2015; Antony et al., 2019; Soluri et al., 2020). Once the domainome libraries are transferred into systems like phage display for functional screening, they can be used to find protein domains that bind to specific targets (like proteins, DNA, sugars, fats, or enzyme substrates) or that have certain enzyme activities—if the right screening tools are available (Soluri et al., 2020; Puccio et al., 2020).

The whole process is called “interactome-seq” (Soluri et al., 2018; Puccio et al., 2020) and has been used in several research projects in our lab (Fasolo et al., 2019; Patrucco et al., 2015). Building these libraries takes less effort and time—usually under two weeks—compared to traditional large-insert metagenomic libraries, and the process is much easier (Lam et al., 2015).

Each year, two to four undergraduate biotechnology students (pursuing a bachelor’s degree) carry out their internship for their thesis project in our lab. They usually spend one semester in the lab, earning a total of 6 credits or ECTS (European Credit Transfer and Accumulation System). We questioned whether this technique could be taught and learned quickly, and whether it would be suitable for simultaneously running several different, low-cost thesis projects. A group of students (eleven in total) from the bachelor’s program in Biotechnology, assisted by their thesis mentors and older lab mates (master’s and PhD students), have worked to optimize a protocol for the construction and analysis of metagenomics *domainome* libraries (Soluri et al., 2018).

As a result, a general laboratory activity has been defined, divided into a 3-week period, and organized according to the scheme summarized below. A detailed list of instruments, kits and reagents required is provided as Supplementary Material.

## 2 Course structure

### 2.1 Week 1. Metagenomic DNA preparation

Metagenomic DNA (mgDNA) can be directly extracted from environmental samples like soil, air, hot spring water, animal skin, faeces, toilet seats, or landfill soil (Bag et al., 2016). There are many kits and protocols available for this. To build one library, at least 10 μg of mgDNA is needed, which can be hard to get depending on the sample (Soluri et al., 2018). For instance, faeces—a rich source of microbes—can provide up to 10 μg of DNA from 100 mg of material (Claassen et al., 2013). As another option, microbes can be grown in suitable liquid or solid media, and mgDNA can be extracted after collecting the cells.

However, this step can introduce a bias in microbiota diversity, as the culture conditions (such as medium composition, incubation temperature, and oxygen concentration) will significantly influence microbial growth. As a result, the final composition of the microbial population will not accurately represent the true microbiome composition. This factor must be considered when discussing the results and drawing conclusions.

### 2.2 Week 2. Library construction

This part is the most technically challenging. The mgDNA must be randomly fragmented, for example, by mechanical (sonication, nebulization) or enzymatic (nuclease) means (Ribarska et al., 2022). The DNA fragments are then sorted by size, which can be obtained inexpensively by resolving the DNA by agarose gel electrophoresis, cutting a gel slice corresponding to the desired size range (250 bp–1,000 bp), and extracting the DNA from the gel (Soluri et al., 2018). Alternatively, a DNA sizing kit can be used. Purified DNA is then repaired, filled-in, and ligated into the pFILTER vector previously linearized with EcoRV (Soluri et al., 2018). This restriction enzyme creates blunt ends, so the digested plasmid needs to be dephosphorylated after cutting to prevent its self-circularization. The ligase reaction is then transformed into competent *E. coli* and the bacteria first seeded on agar plates containing chloramphenicol as selection. Chloramphenicol selection helps to recover all clones with a DNA insert, regardless of whether it contains a real ORF. Transformants grown on chloramphenicol plates are then transferred to ampicillin plates to “filter” for clones that contain a DNA insert placed in the correct reading frame (ORF), along with the signal peptide and the β-lactamase gene. In a typical experiment, chemical transformation by “heat shock” would provide <1,000 colony forming units (cfu), sufficient for a practical laboratory course or a small thesis work (von der Haar, 2019). If a higher complexity of the library is desired, for example, for projects involving the use of Next-Generation Sequencing (NGS) technologies, we suggest transforming the ligation products by electroporation (1.8 kV, time constant 4–5 ms), as it will easily yield >10^6^ clones (Soluri et al., 2018).

### 2.3 Week 3 and beyond. Data analysis

This part gives more space to creativity. The simplest experiment students can perform is to randomly pick one or few colonies from the plate and sequence the cloned DNA fragment. The sequences will then be analysed using Nucleotide Blast (BLASTN) to identify the host organism, followed by *in silico* translation and analysis with Protein Blast (BLASTP) to confirm that the selected genomic fragment is protein-coding DNA (Camacho et al., 2009). At this stage, students can be guided to use various other tools, such as performing phylogenetic analyses (Jacques et al., 2023) or predicting protein solubility (Kyte and Doolittle, 1982) or 3D structure (Jumper et al., 2021), among others.

Since the DNA fragments cloned into pFILTER will be expressed as recombinant proteins fused to a V5 epitope tag for immunodetection, biochemical characterization can be performed using methods such as SDS-PAGE, Western blotting, or mass spectrometry. It will also be possible to sub-clone the DNA fragment into a plasmid suitable for the expression and purification of recombinant proteins for further characterization.

## 3 Representative results

Here we present some representative results of a thesis aimed at exploring a small library of metagenomic DNA from the intestinal microbiome, in search of DNA sequences encoding novel proteins. Most of the work was conducted by a single student (Morra, 2021), although all co-authors, with their previous work, contributed to the optimization of the whole procedure (Bovio, 2019; Ivagnes, 2019; Gandini, 2019; Ottolini, 2019; Marradi, 2020; Maraschi, 2024). A schematic overview of the student’s project is provided in Figure 1. The mgDNA was extracted from murine faeces using a commercial kit (QIAGEN cat. 51804). Ten micrograms of mgDNA were randomly fragmented using a tip sonicator, applying a 15 s pulse and 10% maximum amplitude.

**FIGURE 2:**
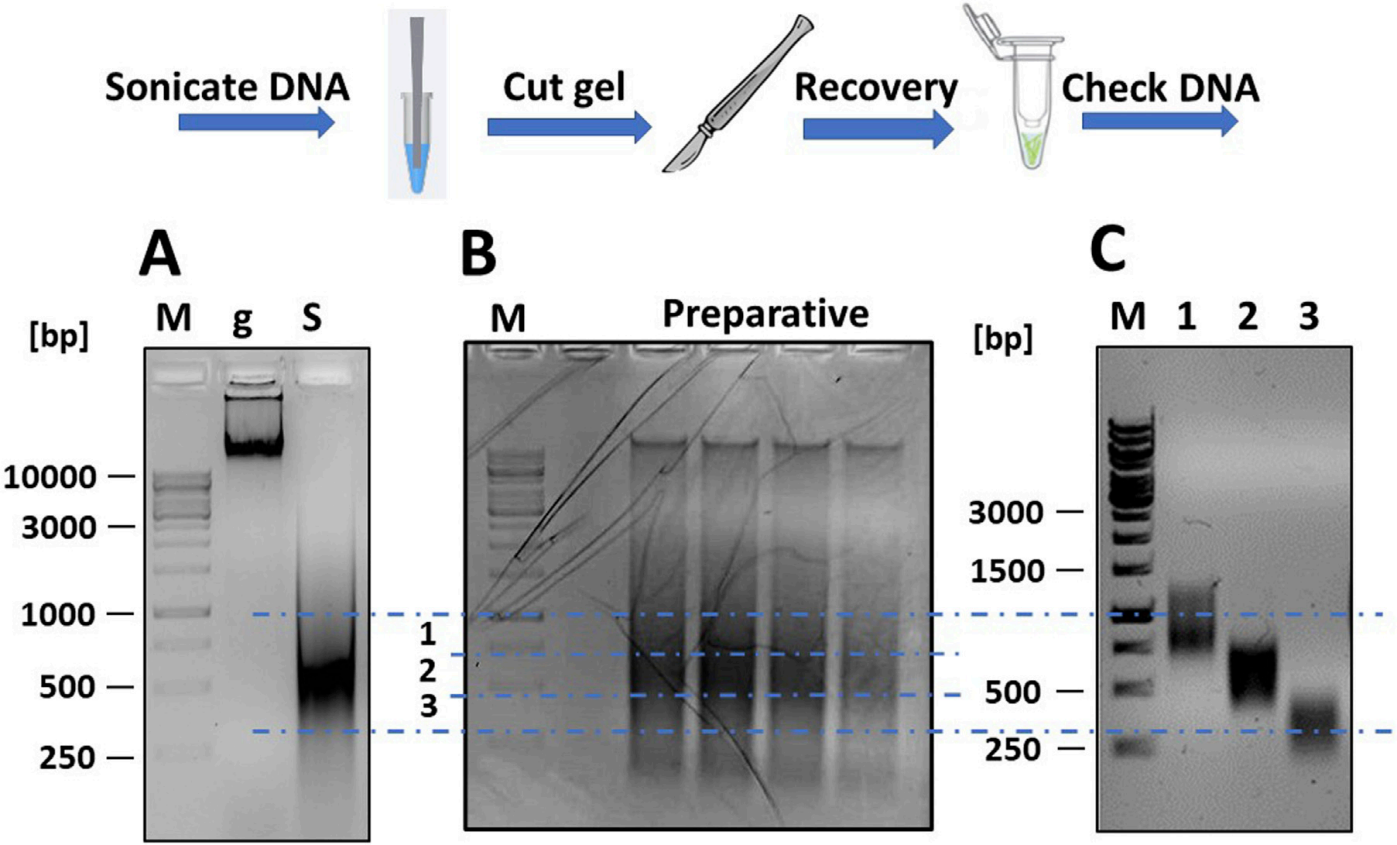
Fragmentation of mgDNA and purification of DNA into the desired size range. **(A)** Approximately 10 μg of mgDNA (lane “g”) are fragmented into 250–1,000 bp fragments by sonication (lane “S”) and checked by gel electrophoresis. **(B)** The sonicated DNA is loaded onto a preparative agarose gel, placed over a blue LED transilluminator, and the gel is cut into several DNA-containing slices of the desired size. **(C)** The pools of DNA fragments are purified from the agarose gel slices and checked again by gel electrophoresis (lane 1: range 250–500 bp; lane 2: range 500–750 bp; lane 3: range 750–1,000 bp; M: DNA ladders).

Fragmentation was verified by agarose gel electrophoresis, running two DNA samples taken before and after sonication, and results are shown in Figure 2A. The non-fragmented DNA appeared as an intense band that partially stuck in the well due to its large size. Following sonication, the DNA appeared as a diffuse smear, ranging in size from <200 to >3,000 bp.

The sonicated DNA was loaded onto a preparative gel (1% agarose in TAE buffer) for purification. After electrophoresis (45 min at 80 V), the gel was examined under a blue light LED transilluminator (Figure 2B). Using a scalpel and referencing the DNA ladders, the gel section containing DNA fragments between 250 and 1,000 bp was divided into three slices and excised. The blue light LED transilluminator was chosen for longer exposure times as it was safer for students and reduced the risk of DNA damage compared to a UV transilluminator. The DNA was then extracted from the gel using a commercial kit (Thermo Fisher Scientific cat. K0832) and re-checked by gel electrophoresis to confirm the correct size range of the DNA fragments (Figure 2C). The DNA was then repaired using a commercial kit for DNA blunting (NEB cat. E1201), as described (Soluri et al., 2018). The DNA fragments were then ligated into the linearized pFILTER vector with a T4 DNA ligase (Thermo Fisher Scientific cat. EL0014) and transformed into chemically competent *E. coli* DH5αF′ cells.

**FIGURE 3:**
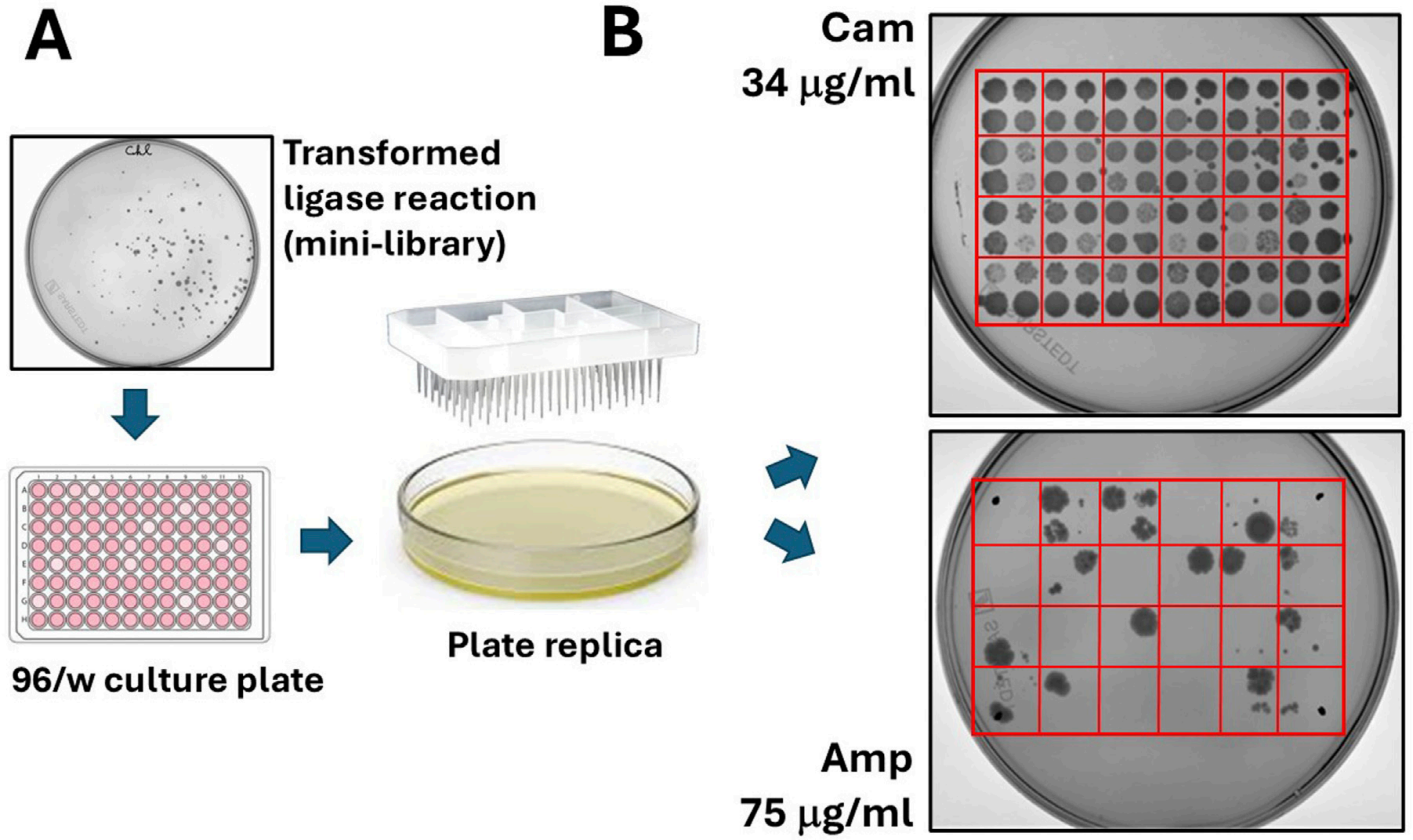
“Filtering” of open reading frames by selection on ampicillin. **(A)** Colonies grown on chloramphenicol agar plates are manually picked and seeded on 96/w plates. From here, colonies are replica plated on chloramphenicol and ampicillin agar dishes. **(B)** A typical result from the replica plating shows 96 colonies (100%) growing on chloramphenicol and much less (typically, only 5–10 colonies) growing on ampicillin.

**FIGURE 4:**
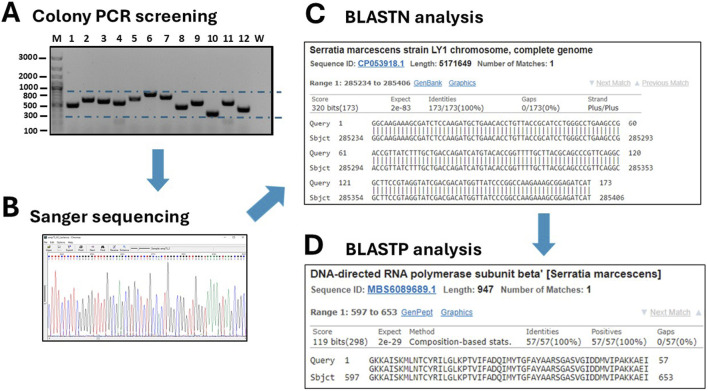
Screening of grown transformants. **(A)** A colony PCR is performed on several randomly picked clones (M: DNA ladders; W: negative control of PCR). **(B)** Positive clones are sequenced by Sanger sequencing and traces are visualized with the Chromas 2.6.6 software. **(C)** DNA sequences are analyses by BLASTN to identify the host organism. **(D)** After *in silico* translation, the sequences are further analyzed by BLASTP to confirm that they belong to true protein-coding genes or to identify a novel, putative protein.

The bacteria were plated onto a 15 cm 2xTY/Cam agar plate (chloramphenicol 34 μg/mL) and incubated at 30°C overnight (O/N). Ninety-six colonies grown on chloramphenicol were selected and inoculated into a 96-well culture plate containing 100 μL of 2xTY/Cam medium. After incubating for 2 hours at 30°C, the colonies were transferred from the 96/w plate onto two 15 cm Petri dishes—one with 34 μg/mL chloramphenicol and the other with 75 μg/mL ampicillin. To do this, we used a 96-well pin replicator, depicted in Figure 3A. Plated colonies were grown O/N at 30°C. As shown in Figure 3B (upper-right panel), all colonies grew on chloramphenicol, while only some of them (around 10–15 clones) grew on ampicillin. We continued analysing 12 bacterial clones: four grown only on chloramphenicol (numbered 1–4), and 8 grown on ampicillin (numbered 5–12). The DNA inserts were amplified by colony PCR by using a pair of external primers and analysed by gel electrophoresis. The sequences of primers (pDAN_filter_sense/anti) are provided in the Supplementary Material. From the image of the gel presented in Figure 4A, it is possible to appreciate the presence of amplicons of different lengths ranging between 300 and 800 bp, suggesting that the various clones contain different DNA inserts.

**FIGURE 5:**
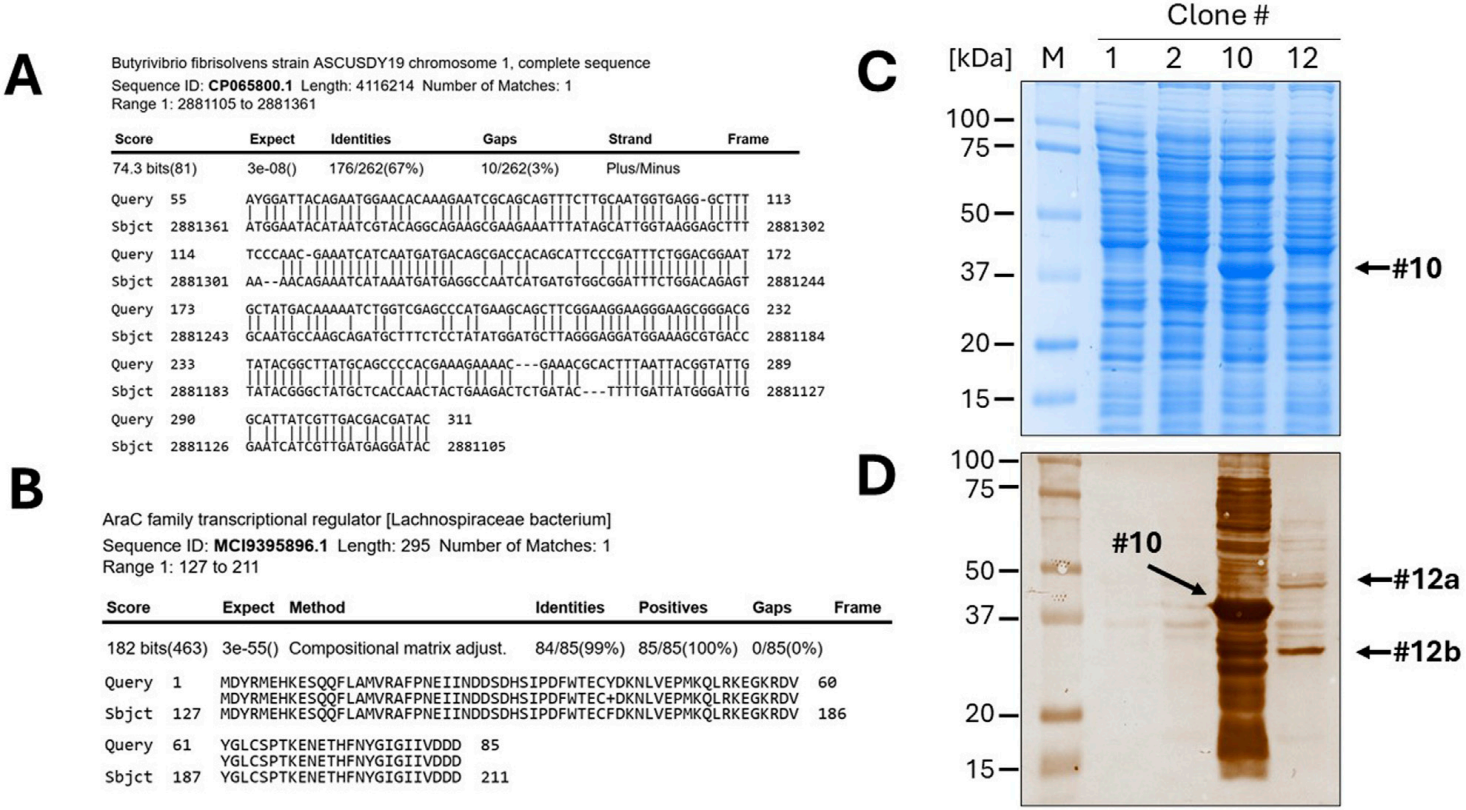
Identification of a novel, unannotated DNA sequence and expression of recombinant proteins. **(A)** The BLASTN analysis of clone 10 revealed only a 67% homology to the *Butyrivibrio fibrisolvens* genome. **(B)** After *in silico* translation and BLASTP, the DNA was identified as a fragment of a gene encoding a member of the AraC family of transcriptional regulator. Gel electrophoresis (SDS-PAGE) of bacterial lysates of the selected clones (1, 2, 10 and 12), followed by Coomassie blue staining **(C)** or Western blotting **(D)**, confirmed the efficient expression of the protein domains fused to β-lactamase.

The twelve clones were cultured in 2 mL of 2xTY medium with chloramphenicol for plasmid extraction, which was performed using a commercial plasmid DNA miniprep kit (Thermo Fisher Scientific cat. K0702). The inserts were then sequenced by Sanger sequencing using either the sense or antisense primer from the colony PCR. DNA traces (shown in Figure 4B) were visualized with the software Chromas version 2.6.6 (Technelysium Ltd.). The start and end of the DNA insert were identified in the obtained sequences, located adjacent to the consensus sequences recognized by EcoRV (GAT_ATC). The sequences were analysed using BLASTN to determine the species origin of the DNA fragments. Subsequently, the nucleotide sequences were *in silico* translated using the Sequence Manipulation Suite (SMS) version 2 (https://www.bioinformatics.org/sms2/). To identify the correct reading frame, the sequence was aligned with the known signal peptide (gca gca agc ggc gcg cat gcc, encoding Ala-Ala-Ser-Gly-Ala-His-Ala) and translated until the first stop codon. Visual confirmation of the inserts was possible by identifying the presence of the secretory leader sequence (L) upstream of the cloning site and the β-lactamase gene downstream, which served as reference markers for correct insert orientation and integration. Finally, BLASTP was used to analyse the resulting amino acid sequences. To provide a detailed illustration of the analysis conducted, the procedure performed for clone 10 is outlined below as an example. The colony PCR screening confirmed the presence of an insert approximately 200 bp in size, which appeared as a 300 bp amplicon on the gel due to the external primers used for PCR (Figure 4B). BLASTN analysis further revealed that a 173 bp segment of the insert was 100% homologous to the *Serratia marcescens* genome (Figure 4C). Although the sonication process was aimed at generating DNA fragments larger than 250 bp, shorter fragments may still be present due to the random nature of shearing and subsequent size selection limitations. Additionally, shorter fragments can sometimes be preferentially amplified or cloned, which may explain the presence of this 173 bp insert. The sequence was then translated *in silico* using the Sequence Manipulation Suite (SMS) tool, following the frame with β-lactamase. A BLASTP search of the amino acid sequence showed the closest match was a DNA-directed RNA polymerase from *S. marcescens* (Figure 4D). The results from clone 12 were particularly interesting. In this case, BLASTN analysis of a 262 bp fragment revealed partial homology (67%) with the genome of *Butyrivibrio fibrisolvens* (Figure 5A). This level of similarity suggests that the analyzed DNA fragment may originate from a microorganism that is phylogenetically related to the *Butyrivibrio* genus (class *Clostridia*), but whose genome may not yet be represented in current databases. Additionally, translation *in silico* of the predicted coding region showed 100% amino acid identity with an AraC-family transcriptional regulator from a *Lachnospiraceae* bacterium, also within the *Clostridia* class (Figure 5B). These findings support the possibility that the fragment is derived from a phylogenetically related, yet potentially unsequenced or underrepresented, microorganism. From this point, it is possible to perform some biochemical assays. An SDS-PAGE and, subsequently, a Western blot were carried out, shown in Figures 5C,D. Protein expression in sample 10 was notably high: a prominent band of approximately 37 kDa was clearly visible even on the Coomassie-stained gel, indicating strong expression (Figure 5C). Western blot analysis confirmed this observation, showing a very intense band at ∼37 kDa, corresponding to the expected size of the expressed fusion protein. This size is consistent with the in-frame cloning of the 173 bp ORF (encoding ∼58 amino acids, ∼6 kDa) fused to the β-lactamase reporter (∼32 kDa). Additional bands at higher molecular weights likely represent protein aggregates, while those at lower molecular weights may correspond to degradation products. Clone 12 also showed detectable expression by Western blotting, although at lower intensity compared to clone 10 (Figure 5D). In this case, two major bands were observed, with apparent sizes of ∼45 kDa and ∼35 kDa, respectively. The ORF length of 262 bp encodes a peptide of ∼10 kDa, which, when fused to β-lactamase, results in a predicted fusion protein of ∼42 kDa. Therefore, the upper band likely represents the full-length chimeric protein, while the lower band is consistent with a degradation product. Clones 1 and 2 were loaded as a negative control since they grew only on chloramphenicol but not on ampicillin and were therefore expected to be unable to produce a functional fusion protein. Sanger sequencing of these two clones showed that the DNA insert was not in the correct frame with the β-lactamase, confirming that the filtering process worked well in selecting protein-coding gene domains.

## 4 Discussion

### 4.1 The value of functional metagenomics to life science education

How can undergraduate research projects in functional metagenomics provide valuable training and help meet curriculum standards, particularly in terms of preparing young scientists for the biological research workforce? Genetics, microbiology, biochemistry, and molecular biology are foundational courses in life science programs. Metagenomics illustrates how the genes of one organism are interconnected with those of others, as well as with the entire community, bridging basic sciences and advanced fields such as ecology, health sciences, and industrial biotechnology. This process highlights the importance of understanding the full diversity of life within a single environment and researching genes and organisms in their context. Since metagenomics spans multiple disciplines, it serves as an effective tool for teaching key themes and concepts that are integral to life science education.

By introducing students to functional metagenomics at the introductory level, with a focus on its practical applications, they can gain a clearer understanding of the fundamental concepts across various fields, the connections between them, and the broader impact of scientific advancements. Presenting functional metagenomics this way can inspire talented students to pursue careers in science by showing them that there are intriguing, unresolved questions they can contribute to answering. This approach fosters an experience of science as dynamic and ever evolving.

### 4.2 Functional metagenomics as a model for education-research integration

Students’ research holds great potential to advance the field of functional metagenomics, given the vast amount of knowledge yet to be discovered. For example, many metagenomics projects involve collecting and analysing large numbers of samples to compare microbial communities from different sites with similar environmental conditions (Rebets et al., 2017; Zhang et al., 2021). Imagine a large-scale project with students from around the world, such as a global microbiome analysis. With a simple infrastructure of sampling kits and established processes, students could significantly expand the available data for functional metagenomic analysis. The development of effective data management systems, bioinformatics tools, technical innovations, and advancements in microbiology in the coming years could make student involvement in metagenomic sampling a viable option. Raising awareness and understanding of these opportunities within the biology research and teaching communities is the first step. It will be essential to create frameworks for engaging students in the study of microbial communities, their interactions with other organisms in various environments, and the practical applications of metagenomics. From there, the role of functional metagenomics in Life Science education can evolve and expand, adapting to the needs of both students and researchers.

## Data Availability

The raw data supporting the conclusions of this article will be made available by the authors, without undue reservation.

## References

[B1] Antony F. Deantonio C. Cotella D. Soluri M. F. Tarasiuk O. Raspagliesi F. (2019). High-throughput assessment of the antibody profile in ovarian cancer ascitic fluids. Oncoimmunology 8, e1614856. 10.1080/2162402X.2019.1614856 31428516 PMC6685609

[B2] Bag S. Saha B. Mehta O. Anbumani D. Kumar N. Dayal M. (2016). An improved method for high quality metagenomics DNA extraction from human and environmental samples. Sci. Rep. 6, 26775. 10.1038/srep26775 27240745 PMC4886217

[B3] Berini F. Casciello C. Marcone G. L. Marinelli F. (2017). Metagenomics: novel enzymes from non-culturable microbes. FEMS Microbiol. Lett. 364, fnx211. 10.1093/femsle/fnx211 29029060

[B4] Bovio A. (2019). Utilizzo del vettore plasmidico pFILTER/TA per la selezione di domini proteici da DNA genomico batterico. Bachelor’s thesis. Vercelli: Università del Piemonte Orientale.

[B5] Camacho C. Coulouris G. Avagyan V. Ma N. Papadopoulos J. Bealer K. (2009). BLAST+: architecture and applications. BMC Bioinforma. 10, 421. 10.1186/1471-2105-10-421 PMC2803857 20003500

[B6] Caudai C. Galizia A. Geraci F. Le Pera L. Morea V. Salerno E. (2021). AI applications in functional genomics. Comput. Struct. Biotechnol. J. 19, 5762–5790. 10.1016/j.csbj.2021.10.009 34765093 PMC8566780

[B7] Claassen S. du Toit E. Kaba M. Moodley C. Zar H. J. Nicol M. P. (2013). A comparison of the efficiency of five different commercial DNA extraction kits for extraction of DNA from faecal samples. J. Microbiol. Methods 94, 103–110. 10.1016/j.mimet.2013.05.008 23684993 PMC5809576

[B8] D’Angelo S. Velappan N. Mignone F. Santoro C. Sblattero D. Kiss C. (2011). Filtering “genic” open reading frames from genomic DNA samples for advanced annotation. BMC Genomics 12 (Suppl. 1), S5. 10.1186/1471-2164-12-S1-S5 PMC3223728 21810207

[B9] Fasolo F. Patrucco L. Volpe M. Bon C. Peano C. Mignone F. (2019). The RNA-binding protein ILF3 binds to transposable element sequences in SINEUP lncRNAs. FASEB J. 33, 13572–13589. 10.1096/fj.201901618RR 31570000 PMC6894054

[B10] Fuhrmeister E. R. Larson J. R. Kleinschmit A. J. Kirby J. E. Pickering A. J. Bascom-Slack C. A. (2021). Combating antimicrobial resistance through student-driven research and environmental surveillance. Front. Microbiol. 12, 577821. 10.3389/fmicb.2021.577821 33679626 PMC7931799

[B11] Gandini L. (2019). Sviluppo di una piattaforma per il “filtering” di open reading frames da DNA genomico batterico. Bachelor’s thesis. Vercelli: Università del Piemonte Orientale.

[B12] Ginnan N. Bordenstein S. R. (2023). It is time to authenticate the Microbiome Sciences with accredited educational programs and departments. PLOS Biol. 21, e3002420. 10.1371/journal.pbio.3002420 38060452 PMC10703218

[B13] Gourlay L. J. Peano C. Deantonio C. Perletti L. Pietrelli A. Villa R. (2015). Selecting soluble/foldable protein domains through single-gene or genomic ORF filtering: structure of the head domain of Burkholderia pseudomallei antigen BPSL2063. Acta Crystallogr. D. Biol. Crystallogr. 71, 2227–2235. 10.1107/S1399004715015680 26527140

[B14] Heger A. Holm L. (2003). Exhaustive enumeration of protein domain families. J. Mol. Biol. 328, 749–767. 10.1016/s0022-2836(03)00269-9 12706730

[B15] Heller D. M. Sivanathan V. Asai D. J. Hatfull G. F. (2024). SEA-PHAGES and SEA-GENES: advancing virology and science education. Annu. Rev. Virol. 11, 1–20. 10.1146/annurev-virology-113023-110757 38684129

[B16] Hieter P. Boguski M. (1997). Functional genomics: it’s all how you read it. Science 278, 601–602. 10.1126/science.278.5338.601 9381168

[B17] Ivagnes V. (2019). Ingegnerizzazione di un vettore plasmidico compatibile con il metodo “TA cloning” per il clonaggio e la selezione di librerie di open reading frames. Bachelor’s thesis. Vercelli: Università del Piemonte Orientale.

[B18] Jacques F. Bolivar P. Pietras K. Hammarlund E. U. (2023). Roadmap to the study of gene and protein phylogeny and evolution—a practical guide. PLOS ONE 18, e0279597. 10.1371/journal.pone.0279597 36827278 PMC9955684

[B19] Jumper J. Evans R. Pritzel A. Green T. Figurnov M. Ronneberger O. (2021). Highly accurate protein structure prediction with AlphaFold. Nature 596, 583–589. 10.1038/s41586-021-03819-2 34265844 PMC8371605

[B20] Jurkowski A. Reid A. H. Labov J. B. (2007). Metagenomics: a call for bringing a new science into the classroom (while it’s still new). CBE Life Sci. Educ. 6, 260–265. 10.1187/cbe.07-09-0075 18056294 PMC2104496

[B21] Kyte J. Doolittle R. F. (1982). A simple method for displaying the hydropathic character of a protein. J. Mol. Biol. 157, 105–132. 10.1016/0022-2836(82)90515-0 7108955

[B22] Lam K. N. Cheng J. Engel K. Neufeld J. D. Charles T. C. (2015). Current and future resources for functional metagenomics. Front. Microbiol. 6, 1196. 10.3389/fmicb.2015.01196 26579102 PMC4625089

[B23] Land M. Hauser L. Jun S. R. Nookaew I. Leuze M. R. Ahn T. H. (2015). Insights from 20 years of bacterial genome sequencing. Funct. Integr. Genomics 15, 141–161. 10.1007/s10142-015-0433-4 25722247 PMC4361730

[B24] Maraschi L. (2024). Costruzione di una libreria di domini proteici espressi dal genoma di SARS-CoV-2. Bachelor’s thesis. Vercelli: Università del Piemonte Orientale.

[B25] Marradi D. (2020). Utilizzo della tecnica “orf filtering” per lo studio e caratterizzazione di nuove specie batteriche. Bachelor’s thesis. Vercelli: Università del Piemonte Orientale.

[B26] Morra M. (2021). Sviluppo di una piattaforma per il “filtering” di Open Reading Frames da DNA metagenomico. Bachelor’s thesis. Vercelli: Università del Piemonte Orientale.

[B27] Muth T. R. Caplan A. J. (2020). Microbiomes for all. Front. Microbiol. 11, 593472. 10.3389/fmicb.2020.593472 33281791 PMC7688743

[B28] Ottolini G. (2019). “Filtering” di Open Reading Frames da campioni di DNA genomico per annotazioni avanzate. Bachelor’s thesis. Vercelli: Università del Piemonte Orientale.

[B29] Patrucco L. Peano C. Chiesa A. Guida F. Luisi I. Boria I. (2015). Identification of novel proteins binding the AU-rich element of alpha-prothymosin mRNA through the selection of open reading frames (RIDome). RNA Biol. 12, 1289–1300. 10.1080/15476286.2015.1107702 26512911 PMC4829324

[B30] Pouresmaeil M. Azizi-Dargahlou S. (2023). Factors involved in heterologous expression of proteins in *E. coli* host. Arch. Microbiol. 205, 212. 10.1007/s00203-023-03541-9 37120438 PMC10148705

[B31] Puccio S. Grillo G. Consiglio A. Soluri M. F. Sblattero D. Cotella D. (2020). InteractomeSeq: a web server for the identification and profiling of domains and epitopes from phage display and next generation sequencing data. Nucleic Acids Res. 48, W200–W207. 10.1093/nar/gkaa363 32402076 PMC7319578

[B32] Qin J. Li R. Raes J. Arumugam M. Burgdorf K. S. Manichanh C. (2010). A human gut microbial gene catalogue established by metagenomic sequencing. Nature 464, 59–65. 10.1038/nature08821 20203603 PMC3779803

[B33] Rebets Y. Kormanec J. Luzhetskyy A. Bernaerts K. Anné J. (2017). Cloning and expression of metagenomic DNA in streptomyces lividans and subsequent fermentation for optimized production. Methods Mol. Biol. 1539, 99–144. 10.1007/978-1-4939-6691-2_8 27900687

[B34] Ribarska T. Bjørnstad P. M. Sundaram A. Y. M. Gilfillan G. D. (2022). Optimization of enzymatic fragmentation is crucial to maximize genome coverage: a comparison of library preparation methods for Illumina sequencing. BMC Genomics 23, 92. 10.1186/s12864-022-08316-y 35105301 PMC8805253

[B35] Soluri M. F. Puccio S. Caredda G. Edomi P. D’Elios M. M. Cianchi F. (2020). Defining the *Helicobacter pylori* disease-specific antigenic repertoire. Front. Microbiol. 11, 1551. 10.3389/fmicb.2020.01551 32849324 PMC7396715

[B36] Soluri M. F. Puccio S. Caredda G. Grillo G. Licciulli V. F. Consiglio A. (2018). Interactome-seq: a protocol for domainome library construction, validation and selection by phage display and next generation sequencing. J. Vis. Exp., 56981. 10.3791/56981 30346377 PMC6235410

[B37] Terrón-González L. Genilloud O. Santero E. (2014). “Potential and limitations of metagenomic functional analyses,” in Metagenomics, methods, applications and perspectives (New York: Nova Publishers), 1–43.

[B38] Tiessen A. Pérez-Rodríguez P. Delaye-Arredondo L. J. (2012). Mathematical modeling and comparison of protein size distribution in different plant, animal, fungal and microbial species reveals a negative correlation between protein size and protein number, thus providing insight into the evolution of proteomes. BMC Res. Notes 5, 85. 10.1186/1756-0500-5-85 22296664 PMC3296660

[B39] von der Haar T. (2019). Preparation and transformation of competent *E. coli* cells (CCMB80 method). 10.17504/protocols.io.hayb2fw

[B40] Wiltschi B. Cernava T. Dennig A. Galindo Casas M. Geier M. Gruber S. (2020). Enzymes revolutionize the bioproduction of value-added compounds: from enzyme discovery to special applications. Biotechnol. Adv. 40, 107520. 10.1016/j.biotechadv.2020.107520 31981600

[B41] Zhang L. Chen F. Zeng Z. Xu M. Sun F. Yang L. (2021). Advances in metagenomics and its application in environmental microorganisms. Front. Microbiol. 12, 766364. 10.3389/fmicb.2021.766364 34975791 PMC8719654

