# Supplementary material for: A non-hypothesis-driven practical laboratory activity on functional metagenomics: “fishing” protein-coding DNA sequences from microbiomes
Source: Front Bioeng Biotechnol. 2025 May 20;13:1602982. doi: 10.3389/fbioe.2025.1602982 (PMC12131325; doi:10.3389/fbioe.2025.1602982)
Supplement: Supplementary file 1 [file Table1.docx]

# Supplemental information

## Key resource table

| **Reagent or Resource** | **Source(s)** | **We used…** |
| --- | --- | --- |
| ***Bacterial strains*** | | |
| *E. coli* DH5α (chemically competent)  Genotype: F^–^ φ80*lac*ZΔM15 Δ(*lac*ZYA-*arg*F)U169 *rec*A1 *end*A1 *hsd*R17(r_K_^–^, m_K_^+^) *pho*A *sup*E44 λ^–^*thi*-1 *gyr*A96 *rel*A1 | Many suppliers | we make our own competent cells |
| ***Oligonucleotides*** | | |
| Primer fwd pDAN_filter_sense GCAGCAAGCGGCGCGCATGCC | Many suppliers | Integrated DNA technologies (IDT) |
| Primer rev pDAN_filter_anti GGGATTGGTTTGCCGCTAGC | Many suppliers | Integrated DNA technologies (IDT) |
| ***Chemicals and Reagents*** | | |
| 2xYT medium | Many suppliers | Sigma-Aldrich cat. Y2377 |
| Ampicillin | Many suppliers | Sigma-Aldrich cat. A9518 |
| Chloramphenicol | Many suppliers | Sigma-Aldrich cat. C1919 |
| Coomassie Blue | Many suppliers | Sigma-Aldrich cat. B0149 |
| Deoxyribonucleotide triphosphates (dNTPs) set | Many suppliers | Thermo Fisher Scientific cat. R0181 |
| Taq DNA Polymerase (5 U/μl) | Many suppliers | Thermo Fisher Scientific cat. EP0701 |
| EcoRV | Many suppliers | New England Biolabs (NEB) cat. R0195 |
| DNA Ladder | Many suppliers | GeneRuler 1 kb DNA Ladder (Thermo Fisher Scientific) cat. SM0311 |
| Protein Ladder | Many suppliers | PageRuler^TM^ prestained Protein Ladder (Thermo Fisher Scientific) cat. 26616 |
| T4 DNA ligase (5 U/μl) | Many suppliers | Thermo Fisher Scientific cat. EL0014 |
| V5 Tag Monoclonal Antibody (E10/V4RR) | Thermo Fisher Scientific | MA5-15253 |
| Goat Anti-Mouse IgG – Alkaline Phosphatase | Many suppliers | Sigma-Aldrich cat. A2179 |
| *Commercial Kits & Instruments/Materials* | | |
| BigDye™ Terminator v1.1 Cycle Sequencing Kit | Thermo Fisher Scientific | 4337450 |
| Gel Extraction and DNA Cleanup Kit | Many suppliers | Thermo Fisher Scientific GeneJET cat. K0832 |
| Plasmid Miniprep kit | Many suppliers | Thermo Fisher Scientific GeneJET cat. K0702 |
| Blunting Kit | Many suppliers | New England Biolabs (NEB) cat. E1201 |
| PCR clean-up kit | Many suppliers | Thermo Fisher Scientific cat. K310001 |
| Stool DNA Metagenome extraction kit |  | QIAGEN cat. 51804 |
| ***Equipment and Instruments*** | | |
| Benchtop Centrifuge | Many suppliers | Eppendorf cat. 5804 R |
| Imaging system | Many suppliers | ChemiDoc Bio-Rad cat. 12003153 |
| Water bath | Many suppliers | Grant model CB2 5QZ |
| UV/Vis spectrophotometer | Many suppliers | Thermo Scientific NanoDrop Cat # ND2000 |
| Orbital shaker | Many suppliers | Jeio tech Cat # IS-971R |
| Plate replicator, 96-well | Sigma-Aldrich | cat. Z370819 |
| Tip sonicator | Many suppliers | Bandelin Sonopuls HD 2070 cat. 10548351 (Fisher scientific) |
| Thermal cycler | Many suppliers | LifePro Bioer |
| Sanger DNA sequencer |  | SeqStudio Genetic Analyzer (Thermo Fisher Scientific) cat. A35644 |
| Electroporator | Eppendorf 2510 | 4307 000.658 |
| *Software, algorithms, and web resources* | | |
| Chromas v2.6.6 | <https://technelysium.com.au/wp/chromas/> | |
| BLAST (Sayers et al., 2022) | <https://blast.ncbi.nlm.nih.gov/Blast.cgi> | |
| The sequence manipulation suite v2 (Stothard, 2000) | <https://www.bioinformatics.org/sms2/index.html> | |

Sayers, E. W., Bolton, E. E., Brister, J. R., Canese, K., Chan, J., Comeau, D. C., … Sherry, S. T. (2022). Database resources of the national center for biotechnology information. *Nucleic Acids Research*, *50*(D1), D20–D26. doi: 10.1093/nar/gkab1112

Stothard, P. (2000). The sequence manipulation suite: JavaScript programs for analyzing and formatting protein and DNA sequences. *BioTechniques*, *28*(6), 1102, 1104. doi: 10.2144/00286ir01
